# Pulsed radio wave as a sustainable control technology to improve indoor air quality

**DOI:** 10.1038/s41598-024-61754-4

**Published:** 2024-05-13

**Authors:** G. S. N. V. K. S. N. Swamy Undi, Cheramangalath Balan Ramya, Srikanth Sola, Radhica Kanniganti, Kiran Shinde

**Affiliations:** Research and Development Group, Devic Earth Private Limited, First Floor Sai Lakshmi Industries, Bangalore, Karnataka 560067 India

**Keywords:** Indoor air pollution, Particulate matter, Control technologies, Climate sciences, Environmental sciences

## Abstract

The need for technologies that can clean the air indoors has grown in pace with the rise in outside pollution. Maintaining interior environment adaptability requires a permanent air purification system that may be utilized to control PM_2.5/10_. In addition to more traditional methods of air purification, developing advanced control systems that effectively reduce PM levels sustainably is necessary. Pulsed radio waves may expedite the dry deposition of particles having aerodynamic dimensions of less than 30 µm. The charging and coagulation processes are evaluated in an indoor restricted environment. Experimental results reveal a similar pattern to the Monte Carlo models. Distribution of charge due to the nature of the charging environment increases the coagulation rate. Contained experimental testing confirms the filtering system functions as expected, corroborated by the present research. Daily average levels of PM_2.5_ and PM_10_ were lowered by between 55 and 41% according to a study done in three indoor settings using the control technology. Research conducted throughout all seasons showed that the approach was consistently efficient in reducing PM_2.5/10_. It was shown that PM concentrations could be lowered by around 45 percent using pulse radio wave technology, leading to this conclusion. The use of electromagnetic waves (EM waves) to eliminate air pollution has been suggested as a radical new approach. Considering the limitations of already used strategies, this is of paramount significance while considering solutions to control air pollution.

## Introduction

The adverse effects of inadequate air quality on human health, specifically in terms of respiratory and cardiovascular ailments, as well as untimely mortality, are a substantial global apprehension that presents a serious peril to the welfare of mankind. The importance of air quality with overall human well-being is emphasized by the considerable levels of consumption, namely above 20 m^3^ per day^1–3^. Ensuring the maintenance of ideal air quality levels is of paramount significance, as individuals spend a considerable amount of their time indoors^2,3^. Indoor air pollution ranks among the top five environmental hazards, as stated by the Environmental Protection Agency (EPA)^4^. The interior ambient conditions have a direct impact on the performance of both physical and mental labor. The study’s results suggest that individuals demonstrate improved performance when they are in environments that have adequate indoor air quality. Based on the findings of Lin et al.^5^, extant literature indicates that a 10% rise in discontent is associated with a corresponding decrease in performance of approximately 1%. Moreover, empirical research conducted in the field has provided compelling data supporting a substantial correlation between performance levels and enhanced indoor air quality. Insufficient air circulation and confinement hinder the removal of contaminants from indoor spaces. Because persons generally spend around two-thirds of their daily time indoors, the prevalence of indoor air pollution (IAP) presents a more significant risk and leads to increased health dangers. According to recent research findings, it has been demonstrated that the concentration of air pollutants within interior spaces can be up to tenfold greater than the corresponding levels of outside air pollution. The underlying cause of this phenomenon can be attributed to the fact that confined spaces have a greater propensity for accumulating potential contaminants in comparison to open environments^6^.

Compared to external sources of pollution, such as water and soil, which individuals can avoid ingesting, indoor air pollution poses a far more critical concern. In addition to many other aspects, exposure to inferior air quality is known to substantially elevate the probability of encountering health-related issues. Exposure to hazardous compounds, even for brief periods, can lead to a range of adverse effects on health, including sick-building syndrome, cardiovascular diseases, headaches, and fatigue. There is a recognized necessity for a cost-effective and highly efficient indoor air filtration system that possesses strong capabilities to effectively reduce pollution levels and guarantee the maintenance of a healthy indoor environment. Numerous study groups have undertaken investigations that yield significant empirical support for the association between indoor air pollution and respiratory illness^7–10^. Based on many studies conducted by^10–12^ have demonstrated that insufficient indoor air quality can result in immediate detrimental health consequences, including headaches, nausea, and exhaustion. Chang et al.^13^ assert that tiny particulate matter (PM) possesses the capacity to traverse the pulmonary barrier and get access to the circulatory system, while coarse PM predominantly infiltrates the lungs. Roth^14^ presents empirical evidence indicating a potential association between particulate matter and a decline in human cognitive functions. The prevalence of air pollution in office buildings is a frequent phenomenon, characterized by many sources of indoor contaminants that might have equally harmful effects in some cases. Certain architectural constructions may demonstrate elevated amounts of air pollution in comparison to their immediate surroundings. The examination and consequences of this type of pollution are further convoluted by the substantial disparities in geographical circumstances. Sick building syndrome (SBS), building-related illness (BRI), and environmental sensitivity are terms employed to describe health concerns that have been linked to inadequate indoor air quality (IAQ). Distinguishing Sick Building Syndrome (SBS) from other disorders associated with buildings entails identifying certain differences. A significant proportion of the symptoms commonly linked with sick building syndrome exhibit a lack of specificity, as they cannot be conclusively attributed to a particular illness or identifiable source of contamination. Numerous ailments have a significant impact on those residing in unsanitary environments. Brain injury (BRI) is a pathological condition characterized by the presence of clinically recognized causes. The illnesses commonly discussed in medical literature encompass hypersensitivity pneumonitis, humidifier fever, asthma, and allergic rhinitis, all of which exhibit a distinct constellation of symptoms.

Current strategies focused on improving indoor air quality encompass several methods such as enhanced ventilation, source control, and the utilization of air purifiers employing filtration mechanisms. However, it is worth noting that the first two solutions display a higher level of space efficiency and exhibit commendable performance in contemporary architectural designs. In general, traditional air purifiers employ a blend of HEPA filters, activated carbon filters, and ionizers to efficiently purify the air within a confined area. While these technologies can be advantageous, they are also bound by certain limitations such as restricted coverage and substantial maintenance costs.

The utilization of pulsed radio wave technology in Effluent Treatment Plants (ETP) and Sewage Treatment Plants (STP) for the disinfection of wastewater by eliminating pathogenic bacteria can be traced back to its development in the 1970s and subsequent introduction to the market in the 1990s. These devices enabled the amplification of pollutant charges, expediting the processes of flocculation and sedimentation. However, the utilization of these devices has experienced a notable decrease because they utilized basic pulse patterns, which necessitate considerable power resources and large antennas^15^.

A novel method has been devised to enhance indoor air quality in expansive indoor spaces through the utilization of pulsed radio wave technology. The technology being proposed is the transmission of pre-determined sequences of pulses within the frequency range spanning from 2.4 to 2.5 GHz. The utilization of a circularly polarized Omnidirectional transmitter antenna facilitates the attainment of this objective. The application of this technique within large indoor environments has demonstrated significant efficacy in mitigating the levels of particulate matter pollutants, such as PM_2.5_ and PM_10_, with reductions ranging from 30 to 50%. Filter-based air filtration systems are frequently employed to improve the overall air quality within interior environments. Nevertheless, it is important to acknowledge that these systems possess a restricted scope and entail significant expenses for upkeep. In contrast to pulsed radio-wave technology, which possesses the capability to encompass a significant geographical region without necessitating air cleaning techniques reliant on filtration, the approach presents a starkly contrasting viewpoint. The primary aim of this study was to assess the effectiveness of pulsed radio wave technology in improving the quality of indoor air. More specifically, the focus was on evaluating its impact on the concentrations of PM_2.5_ and PM_10_ in various indoor settings.

## Methodology

The efficacy and functionality of the Pure Skies device were validated through the ongoing surveillance of PM_10_ and PM_2.5_ concentrations at the designated research sites. Baseline data was obtained throughout the initial week without the use of any control system. During this specific period, data regarding the concentrations of PM_10_ and PM_2.5_ air pollutants were collected at regular 5-minute intervals. The purpose of this data collection was to examine and analyze the patterns and trends in air pollution levels. Following the establishment of the baseline, the control system was then placed on-site and initiated, remaining operational for three weeks. During this temporal interval, data was gathered and evaluated employing identical procedures as those employed during the baseline period. Following the conclusion of the implementation phase, the control system underwent deactivation for an additional week to gather data for the aim of assessing the effectiveness of the unit. The section focused on supporting material provides a thorough account of the inquiry, while the results and discussion section provides a detailed explanation of the findings. Moreover, we performed laboratory studies under controlled conditions to authenticate the technology, specifically through Chamber-based analysis.

### Experiments under a controlled atmosphere

To determine particle size and charge distributions using the Aerodynamic Particle Sizer (APS) in a controlled chamber where particulate matter is introduced via a powder feeder while monitoring and maintaining consistent temperature and relative humidity. Powder feeder, APS, Controlled chamber with temperature control (e.g., water circulation system) Humidity, and temperature transmitters particulate matter samples.

### Experiment set-up

The trials were conducted in a 1.11 cubic meter container with dual walls and a vacuum-sealed enclosure. The enclosure had a frontal panel constructed from plexiglass. The experimental trials will provide the particles with an adequate amount of time for coagulation before they eventually settle due to the unique geometric shape and size of the test chamber. The chamber is outfitted with many apertures, including an inlet for the injection of dust and an exit specifically allocated for sampling. To provide a uniform and constant dispersion of particulate matter, a fan with a 12-inch diameter and a rotational speed of 900 revolutions per minute (rpm) is strategically positioned on the ceiling of the chamber. Furthermore, the chamber is furnished with openings that allow for the insertion of temperature and humidity probes. The experimental configuration employs a humidity and temperature transmitter, namely the Vaisala model 337 HMT, which is documented to have an accuracy of 2K for temperature readings and 5% for humidity measurements. Furthermore, a vacuum pump is attached to the chamber to facilitate the process of cleaning.

During the experiment, a double-walled container composed of mild steel was used. To enhance the transmission of pulsed waves, an optical window made of Plexiglass was integrated into the container. The temperature inside the chamber was controlled using a chiller and a heater. The chiller unit assumes a vital function in the regulation of temperature inside the chamber during testing, as it facilitates the circulation of water that has undergone heating or cooling processes. The ongoing measurement of particulate matter (PM) concentrations was carried out using state-of-the-art sensors, as shown in Table 1 of the supplementary materials.

**Table 1:** Monitoring instruments specifications.

Specifications	TSI	PALAS
Make and model	DUST TRACK 8533	Fidas Frog
Principle used	Light Scattering	Aerosol spectrometer
Range of measurement	0–150,000 µg/m^3^	0–50,000 µg/m^3^
Sampling frequency	Continuous	Continuous
Accuracy	single particles > 1 µm	single particles 0.18–18 µm

The dust used for the study was Dolomite DMT. The Dolomite Test is a widely used technique utilized to ascertain the existence and relative abundance of dolomite inside individual specimens. The testing methodology used dust particles with a maximum size of 30 microns. The focus of this composition is on the compound known as calcium magnesium carbonate.

**Figure 1:**
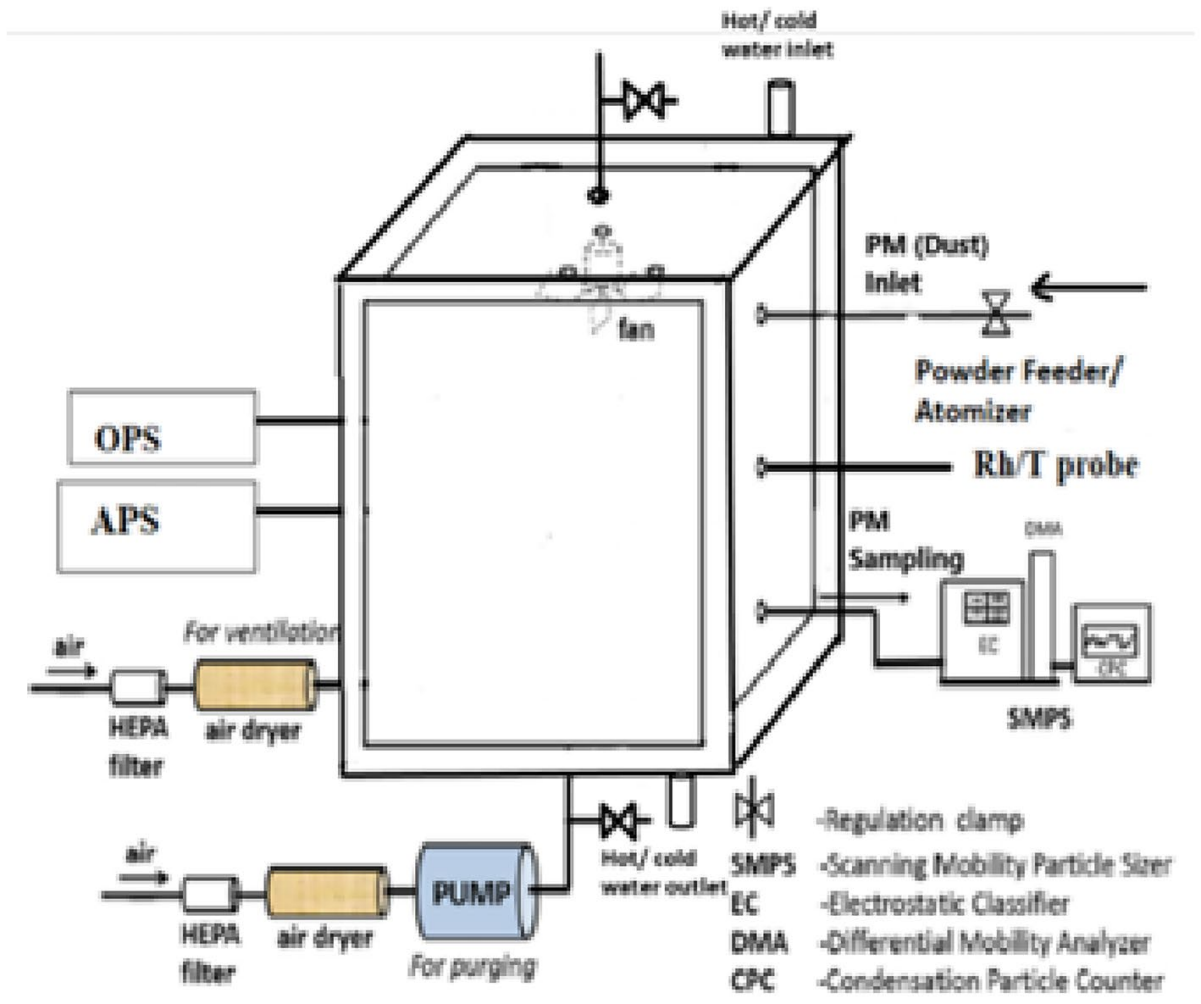
The schematic representation along with all the instruments of the experimental setup to perform chamber-based analysis. Source:^16^.

The subject matter being discussed pertains to dolomite. The investigation detected a maximum particle size of 20 µm. The material has a density of 2.85 grams per cubic centimetre (Fig. 1).

### Preparation of controlled chamber

- Ensure the chamber is thoroughly cleaned and devoid of any residual particles from previous experiments.
- Set up the humidity and temperature transmitters at strategic locations within the chamber to monitor conditions.

### Chamber conditioning

- Start the water circulation system to establish and maintain the desired temperature inside the chamber.
- Set the system to heat or cool water as necessary to achieve and sustain the specified temperature range.

### Powder feeder setup

- Install and calibrate the powder feeder according to manufacturer guidelines.
- Ensure proper connectivity and functionality of the feeder within the controlled chamber.

### Particulate matter introduction

- Begin introducing the desired particulate matter samples into the chamber using the powder feeder.
- Monitor and control the rate of introduction for consistent particle dispersion within the chamber.

### APS measurement setup

- Calibrate the Aerodynamic Particle Sizer (APS) according to its standard operating procedures.
- Position the APS appropriately within the chamber for particle size and charge distribution measurements.

### Data collection

- Start the APS measurement process to collect data on particle size and charge distributions.
- Record measurements at defined intervals, ensuring a sufficient duration for reliable data collection.

### Temperature and humidity monitoring

- Continuously monitor temperature and relative humidity levels using the transmitters within the chamber. The temperature measured during the study varied in range from 27 °C to 33 °C and the RH was about 45% to 60%.
- Record the readings at regular intervals throughout the experiment.

### Controlled environment maintenance

- Ensure the water circulation system maintains a steady and controlled temperature within the specified range.
- Adjust settings if necessary to maintain consistent environmental conditions.

### Experiment termination

- Stop the powder feeder once the desired amount of particulate matter has been introduced.
- Conclude APS measurements and ensure all necessary data is recorded.

### Data analysis

- Analyze APS data to determine particle size and charge distributions.
- Correlate findings with temperature and humidity data to assess potential effects on particle behavior.

Field studies, often known as field research or fieldwork, refer to a research method that involves collecting data and conducting observations in real order to establish a reference point, the air quality is assessed over 14 days under normal operational conditions. The initial air quality is contrasted with the air quality assessed throughout the intervention phase, which spans 2–3 weeks after the installation of the device at the designated location.

The focus of investigation in this study pertains to the geographical setting in which the research is conducted. The research was carried out in three distinct indoor environments, namely: (1) an office setting, (2) a department shop, and (3) an indoor multiplex theater.

The primary research location was a privately owned office facility situated inside an urban setting. The metropolitan area under consideration is characterized by high population density and is known for its significant levels of activity and pollution. Specifically, the region has consistently elevated levels of PM_2.5_ and PM_10_ pollutants, with typical concentrations ranging from 80 to 100 g/m^3^ for 24 h.

A department store used the second location. The inside air quality of the retail shop was constantly poor because of its proximity to a nearby arterial road.

The third location had an indoor multiplex movie theater. The interior air quality was compromised due to its proximity to the mall parking lot, and the external air quality was also suboptimal. In addition, it should be noted that the absence of fresh air is a common characteristic of many movie theaters due to the recirculation of internal air facilitated by air handling systems.

The implementation of the air pollution control unit. The pulsed radio wave devices are enclosed inside a fiber-reinforced plastic cabinet measuring 2 feet in width, 1 foot in depth, and 3 feet in height. The gadget consists of many components including a power source, a signal source, a circularly polarized transmitter antenna, quartz crystals for signal stability, the Internet of Things (IoT) for communication between our laboratory and the unit, an uninterrupted power supply (UPS), and other electrical safety components. The maximum power consumption of each device is 30 watts.

### Air quality monitoring

One pollution control device unit was placed inside each of the three study locations. Furthermore, at each location, an air quality sensor was deployed to evaluate the real-time air quality. The air quality sensors were positioned at a minimum distance of 30–50 meters from the air pollution control device.

The topic of interest pertains to the monitoring of air quality. Throughout the inquiry, indoor air quality sensors were consistently used to assess the amounts of pollution in various locations. In all our deployments, there exists a minimum distance of 30–50 meters between the air quality sensor and the pollution control equipment.

Air quality sensors, namely TSI and PALAS, were used for field installation and experimental testing. To provide up-to-date information on the quality of air, an indoor monitor was used for the evaluation of air quality. The air quality was monitored using PM_10_ and PM_2.5_ sensors based on the laser scattering principle. The sensors underwent calibration against reference-grade monitors before their operation, as shown in Table 1.

### Study protocol

To assess the levels of particle pollution, a comprehensive analysis was conducted wherein the baseline air quality was measured at all designated locations over two weeks while ensuring that the operational conditions remained representative of usual circumstances. After establishing the initial air quality conditions, the pulsed radio wave technique was used to monitor the air quality parameters (PM_2.5_ and PM_10_) throughout a period ranging from one week to one month. This time was identified as the intervention period. Ensuring that both the baseline and intervention periods occurred within the same season was significant. A comparison was made between the air quality during the intervention period and the air quality during the baseline period. In the first month of the intervention phase, a weekly assessment was conducted to compare pollutant levels between the baseline and intervention periods, to determine the extent of effective pollutant reduction. Following the first month of the technological era, a monthly investigation was conducted to ascertain the overall reduction.

## Mechanism coagulation in the presence of external fields

Simultaneous occurrence of aerosol charging and coagulation in atmospheric processes arises due to the interplay between coagulation particles. The size distribution of aerosols undergoes alterations due to the direct impact of particle coagulation. The quantity of charge that can be quantified from an aerosol is closely correlated with the particle size distributions present. The particle size distribution of an aerosol changes when subjected to a charge field, mostly due to the combined effects of Brownian and electrical coagulation^17^. Changes in the concentration of particles and the geometric mean diameter were observed in line with the hypothesis of electromagnetic fields. The modifications were made in consideration of the initial particle number concentration and the duration of residency. The fundamental tool used for the construction of a mechanism capable of simultaneously charging particles and inducing coagulation is the Monte Carlo technique. This approach also demonstrates that the coagulation process significantly alters the characteristics of particle size when it is present in a charged environment. The equation for the total particle number concentration is derived from the accumulation of airborne particles N = $\iint {\text{n}}({\text{r}};\mathrm{ q})\mathrm{ dr dq}$, function of the particle size (r) and charge distribution in the absence of any external particle sources (q)^18^.

The rate of coagulation, denoted by K, between two charged particles in a system with free molecules can be expressed mathematically as

$$K = k \gamma$$

$k$ is the coagulation rate in the absence of charge; $\gamma$ is the collision rate attributed to electrostatic interaction

$$\gamma = \left\{ {\begin{array}{*{20}l} {e^{{\frac{ - \emptyset }{{kT}}}} } \hfill & { for \,\emptyset \ge 0 \left( {repulsion} \right)} \hfill \\ {1 - \frac{\emptyset }{kT} } \hfill & {for \,\emptyset < 0 \left( {attraction} \right)} \hfill \\ \end{array} } \right.$$

where $\varnothing$ is the interaction potential between two particles at contact,

$$\emptyset = \frac{{e^{2} q1q2}}{{4\pi e_{0} \left( {R1 + R2 } \right)}}$$

The rate of charge (R) acquired by the particles is given by in continuum is given by^17,18^

$$R = Z n \pi R^{2} \left( {\frac{8kT}{{\pi M}}} \right) ^{\frac{1}{2}} \gamma$$

where $Z$ is ionic charge; n is ionic concentration; M is ionic mass.

**Figure 2:**
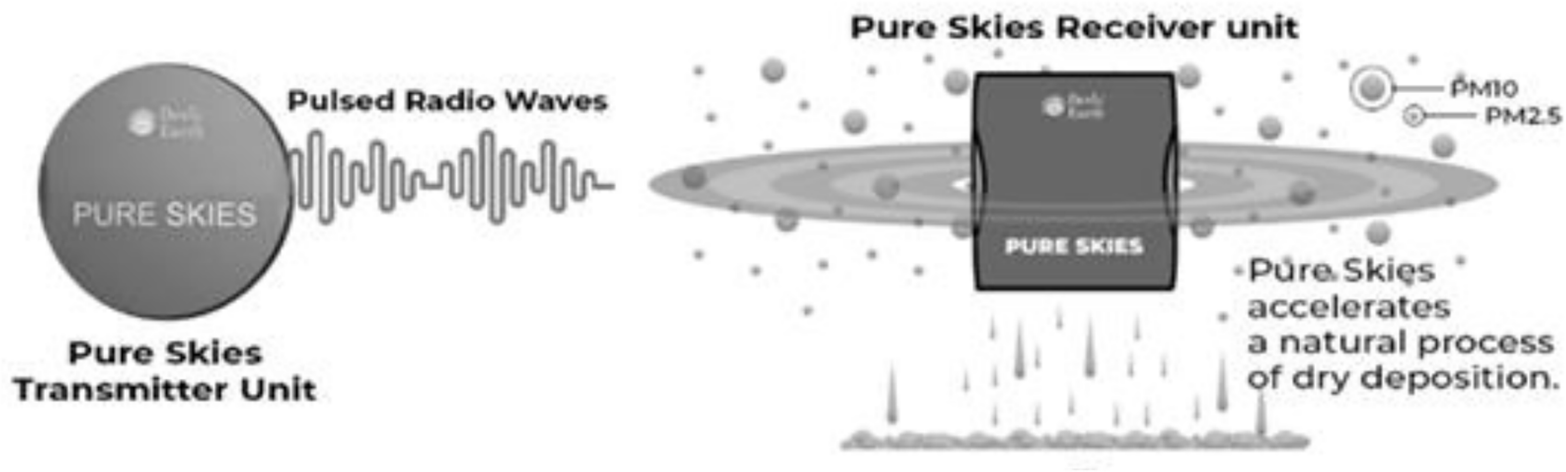
Schematic of Pure Skies, (Created by the authors).

The Pure Skies unit is shown in Fig. 2 on the schematic. Signal Source Generates pulsed radio wave waves. Transmits pulsed radio wave signals in a circular polarization pattern to provide omnidirectional coverage. The Pure Skies device is housed in a fiber-reinforced plastic cabinet. The unit's maximum power consumption is quoted as 30 watts.

The Pure Skies machine transmits predefined sequences of pulsed radio waves in the 2.4 to 2.5 GHz frequency range. These pulsed radio waves are meant to interact with particulate matter (PM_2.5_ and PM_10_) in the indoor environment, causing the particles to charge and coagulate, leading to their dry deposition and thereby improving indoor air quality.

## Results

### Experiments under control environment

**Figure 3:**
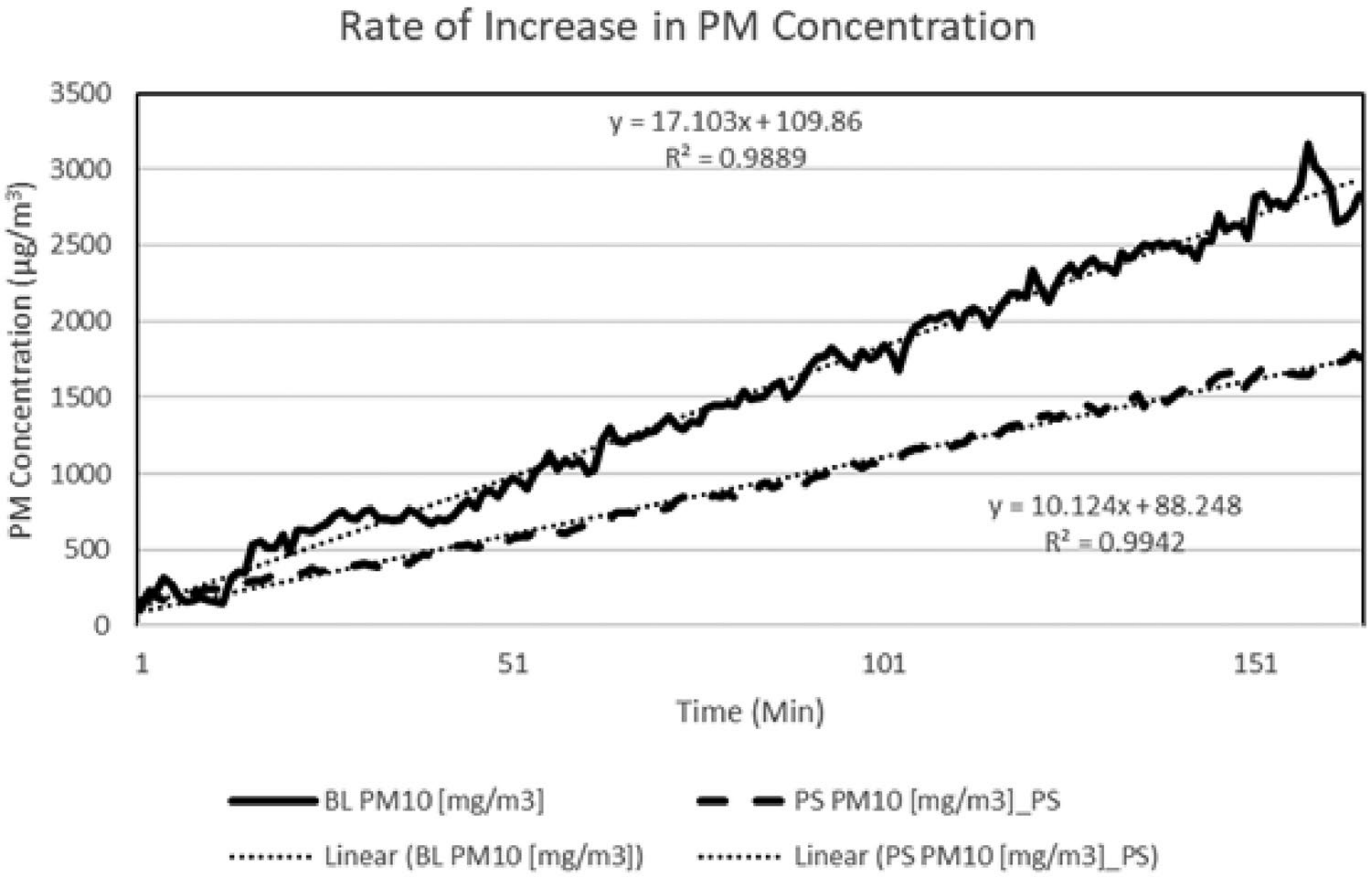
Rate of increase in particulate matter concentration profile with control technology vs baseline.

The quantification of the impact of pulse radio waves on the regulation of particulate matter (PM) concentrations can be determined through the analysis of significant experimental findings. The findings of this investigation demonstrate that radio waves exhibit a discernible capacity to impact the production of particulate matter at its source. The assessment of the control technology's efficacy involves a comparison of the rate at which particulate matter (PM) is generated from the dust dispersion unit. The data reveals a reduction of 41% in the overall rate of PM concentration rise. The generation of particulate matter (PM) displayed a consistent trend, wherein the utilization of control technology resulted in a peak rate of 10 µg/min. Nevertheless, in cases where control technology was not utilized, the documented rate of particulate matter (PM) formation escalated to 17 µg/min. Figure 3 depicts the growth rate of particulate matter (PM) concentration in the presence of control technology, as opposed to the growth rate seen in the absence of such technology, commonly known as the baseline.

**Figure 4:**
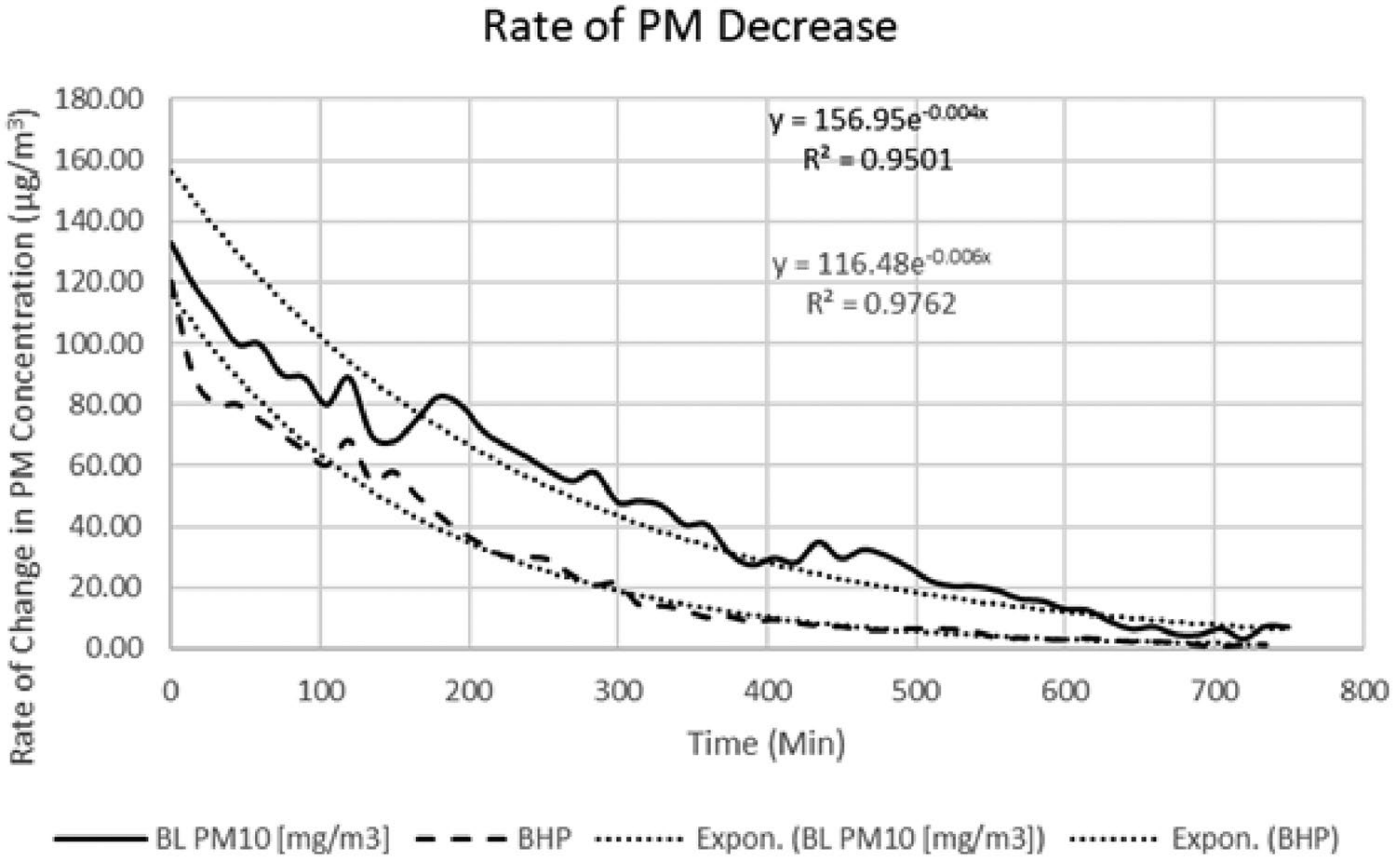
Rate of reduction in PM concentrations with control technology vs baseline.

Figure 4 illustrates the efficacy of several control techniques in managing the concentration of particulate matter (PM). Following the adoption of measures aimed at controlling particulate matter (PM), the emission flow intentionally ceased to evaluate the effectiveness of the control technology in attaining a decrease in PM levels. The results indicated that the decrease in particulate matter (PM) concentrations exhibited a decay rate consistent with a first-order process. Furthermore, it was noted that the rate constant exhibited a value of 0.04 per minute under the baseline condition, which subsequently increased to 0.06 per minute with the implementation of the control technology. This discovery suggests that the introduction of the control technology resulted in a substantial 33 percent increase in the pace of decline.

### Field studies

Field studies, often known as field research or fieldwork, refers to a research method that involves gathering data and conducting observations in a

The data analysis performed to assess the effectiveness of the installed control system within a restricted area revealed a reduction of 45% in PM_10_ levels and a decrease of 65% in PM_2.5_ levels. The information indicated above was reported as follows: Therefore, it can be demonstrated that the utilization of pulsed wave technology is an efficacious strategy for reducing the occurrence of particulate matter in interior environments. The subsequent analysis gives a comparison of the outcomes achieved by the utilization of the technology under consideration, in contrast to the lack thereof.

### An Indoor cinema hall

**Figure 5:**
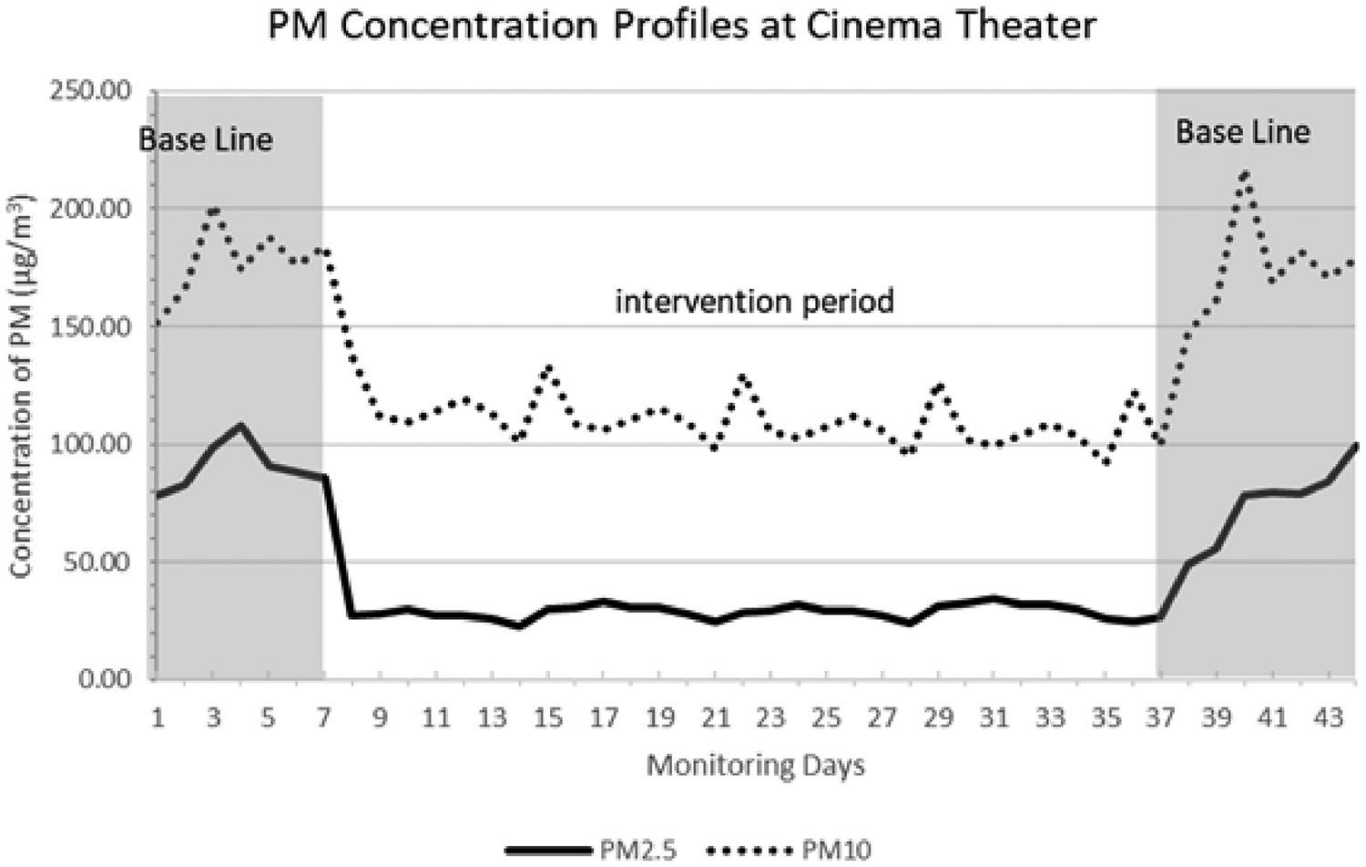
The daily average PM concentration profiles in a cinema theater.

A facility designed to screen films in an enclosed space. Data about the baseline was gathered over a duration of fourteen days. The initial week commenced seven days before the initiation of the intervention phase, whereas the following week occurred seven days after the implementation of the intervention, as seen in Fig. 5. The duration of the intervention phase extended for a period of one month. During the baseline period, the average concentrations of PM_2.5_ and PM_10_ for 24 h were determined to be 83 ± 15 µg/m^3^ and 175 ± 18.5 µg/m^3^, respectively. The implementation of the intervention resulted in a significant decrease of 65% in PM_2.5_ concentrations and 38% in PM_10_ concentrations within the surrounding environment. After a four-week intervention period, it was observed that the mean 24-hour concentrations of PM_2.5_ and PM_10_ decreased to 29 ± 3 µg/m^3^ and 108 ± 11 µg/m^3^, respectively. Based on the findings, it was noted that in the immediate aftermath of the intervention period, the concentrations of PM_2.5_ and PM_10_ levels returned to values comparable to those recorded at the baseline.

### Office space

**Figure 6:**
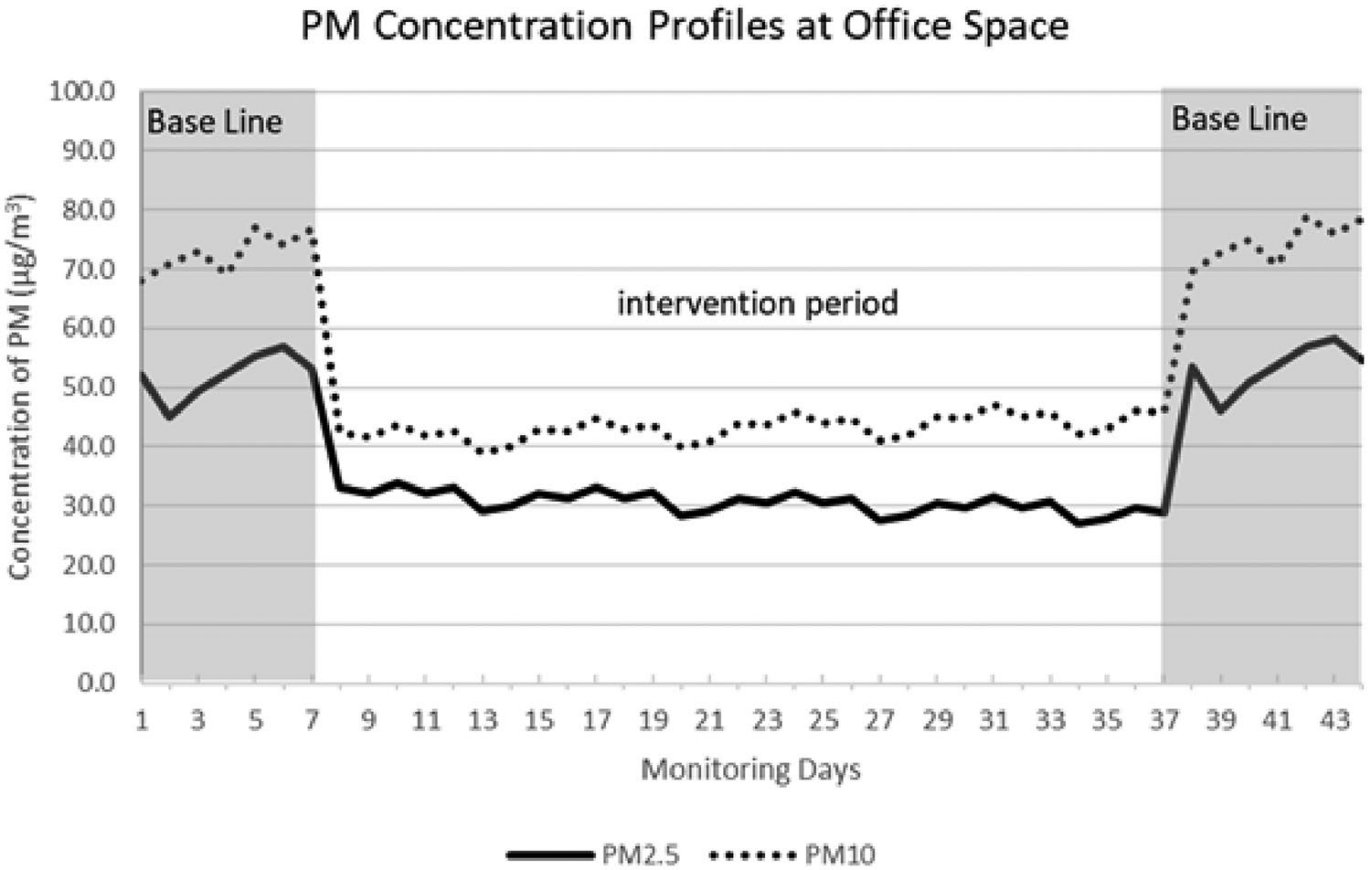
The daily average PM concentration profiles in an office area.

The topic of discussion pertains to the concept of office space. During the intervention period, there was a notable decline in the average concentrations of PM_2.5_ and PM_10_ over 24 h, with reductions of 43% and 41% respectively. The technical infrastructure was operationalized within the workplace setting for a duration of four consecutive weeks. During the initial observation period, the concentration of fine particulate matter (PM_2.5_) was determined to be approximately 53 ± 4 µg/m^3^, whereas the concentration of coarse particulate matter (PM_10_) was documented to be around 73 ± 4 µg/m^3^. After the introduction of the control technology system, often known as the intervention phase, there was a significant reduction in the levels of PM_2.5_ and PM_10_. These concentrations reached values of 30 and 43 micrograms per cubic meter, respectively. After the conclusion of the intervention period, Figure 6 demonstrates an increase in the quantity of particulate matter (PM) inside the specified workplace area.

### Departmental store

**Figure 7:**
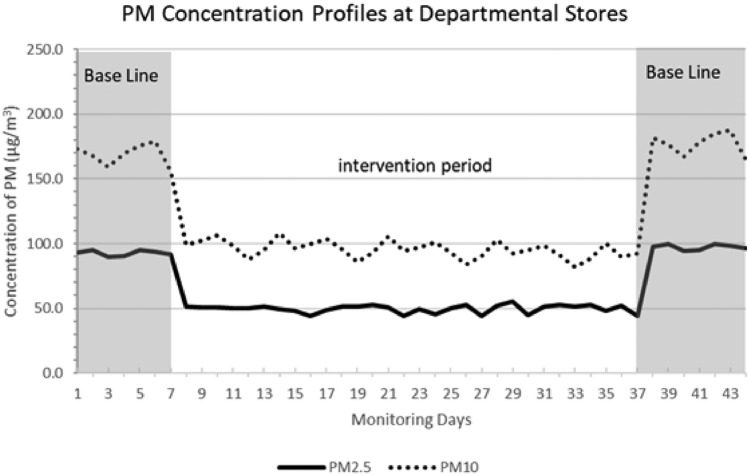
The daily average PM concentration profiles in departmental stores.

The baseline data collection for two weeks was conducted in the department store, as stated earlier. The duration of the intervention session spanned one month. Figure 7 presents a complete analysis of the similarities and differences that occur between PM_2.5_ and PM_10_. The intervention that was executed during that period led to a decrease of 46% and 45% in the 24-hour average concentrations of PM_2.5_ and PM_10_, respectively, in comparison to the initial levels. Moreover, when compared to the baseline, there was a discernible enhancement in air quality as evidenced by the mean readings of PM_2.5_ and PM_10_. During the allocated intervention period, the measured concentrations of PM_2.5_ were reported to be 50 ± 3 µg/m^3^, whereas the concentrations of PM_10_ were measured to be 95 ± 06 µg/m^3^. The initial measurements of PM_10_ were recorded as 175 ± 10 µg/m^3^, whereas the concentrations of PM_2.5_ were determined to be 95 ± 3 µg/m^3^. Upon the trial's culmination, the unit underwent deactivation to evaluate the effectiveness of the system. Following the reinstatement of the levels to their initial values, the effectiveness of the method was confirmed.

### Ambient air quality

The topic of discussion pertains to the quality of ambient air. The atmospheric air quality observed at each of the study sites exhibited characteristics typical of an urban setting. The study conducted in the private office area revealed that the mean concentrations of PM_2.5_ and PM_10_ for 24 h were determined to be 40±14 µg/m^3^ and 67±24 µg/m^3^, respectively. The department store documented the average 24-hour baseline concentrations of PM_2.5_ and PM_10_ as 95±27 µg/m^3^ and 117±30 µg/m^3^, respectively. The numerical values were calculated utilizing the concept of standard deviations. The multiplex cinema theater displayed average concentrations of PM_2.5_ and PM_10_ of 117±9 µg/m^3^ and 208±17 μg/m^3^, respectively, over 24 h. The simultaneous recording of both measures was conducted.

## Discussion

The primary aim of this study was to investigate the impact of pulsed wave technology on indoor air pollutants, primarily focusing on particle pollutants commonly known as PM_2.5_ and PM_10_. The research was conducted within the confines of a corporate office building, a commercial shopping center, and a cinema. Over 24 h, the implementation of pulsed radio wave technology led to a significant reduction in average levels of PM_2.5_ and PM_10_ by approximately 43–65% and 38–45%, respectively, at the specified research sites. The findings of our study indicate that the utilization of pulsed radio wave technology has been recognized as a feasible and effective strategy for reducing particle pollution within indoor settings. Since the early twentieth century, pulsed radio wave technology has been employed for pollution control and the mitigation of specific pollutants in wastewater and sewage. Static electromagnetic fields have been employed for several crucial objectives since the early 1970s. An important milestone in the development of these domains occurred in 1994 when the first patent was secured.

An innovative methodology was developed to efficiently utilize pulsed radio waves to address the issue of air pollution within indoor environments. The technology employed in this study utilizes the intrinsic process of dry deposition as an effective means of removing particulate matter from the environment.

There is a prevailing belief that the pulsed radio waves generated by our technological devices give rise to an electromagnetic field that exhibits spatial non-uniformity, wherein the intensity of the field diminishes as the distance from the antenna increases. The sentence posits the current working hypothesis. According to scientific research, it has been demonstrated that the presence of a weak electromagnetic field can influence suspended particulate matter with an aerodynamic diameter smaller than 20 m. Moreover, this field of research encompasses the manipulation of neutral particles and the formation of transient dipoles, both of which contribute to accelerating the rate of natural dry deposition. As a result, this procedure contributes to the purification of the atmosphere by the efficient elimination of pollutants. The microparticle contaminants demonstrate a phenomenon of net translational migration due to the influence of the dielectrophoretic force,^19,20^ conducted the study.

The wording employed in the article has some variation from that utilized. At each study location, the intervention period was seen to have a considerably longer length compared to the baseline period. Moreover, the instruments employed in the investigation to evaluate air pollutants were air quality monitoring devices that operated on the principle of laser scattering. To ensure accuracy, the air quality monitors were calibrated in advance using a reference-grade monitor.

Improving indoor air quality (IAQ) is a very beneficial undertaking for the building industry, providing substantial economic advantages to society. Even little enhancements to Indoor Air Quality (IAQ) might potentially result in significant reductions in labor expenses because of decreased disease rates and better well-being among building occupants. People residing in a setting marked by substandard indoor air quality may experience a decrease in their productivity and cognitive performance, resulting in substantial negative effects on their general well-being. There is a pressing necessity to promptly implement interventions targeted at mitigating the prevalence of illness and mortality associated with indoor air pollution. Pulsed radio-wave technology possesses a wide coverage area, enabling it to deliver clean air in a highly cost-effective manner per unit of area. Therefore, this technique demonstrates itself as a cost-effective method for enhancing the air quality within expansive structures.

## Conclusion

The variations in the charge of the particle are a result of the stochastic properties that are inherent in the process of charging. The findings of our research are pertinent to particles that experience the phenomenon of obtaining an electrical charge in the presence of a dynamic environment. The positive results of our research emphasize the urgency of promptly implementing this technology across a wide range of extensively utilized infrastructures. The implementation of pollution management technology has been correlated with a reduction in the extent of exposure to particle matter (PM) pollutants, which have been scientifically connected to mortality. Recent research has indicated that the utilization of pulsed radio wave technology has promising capabilities in improving the quality of indoor air inside various interior settings. This study highlights the importance of technology in improving the air quality within large-scale buildings. There exists a necessity for the implementation of more advanced and refined strategies to govern and optimize the administration of atmospheric emissions.

## Data Availability

The datasets generated during and/or analyzed during the current study are available from the corresponding author upon reasonable request.
