# Author Correction: Pulsed radio wave as a sustainable control technology to improve indoor air quality

**DOI:** 10.1038/s41598-024-67338-6

**Published:** 2024-07-24

**Authors:** G. S. N. V. K. S. N. Swamy Undi, Cheramangalath Balan Ramya, Srikanth Sola, Radhica Kanniganti, Kiran Shinde

**Affiliations:** Research and Development Group, Devic Earth Private Limited, First Floor Sai Lakshmi Industries, Bangalore, Karnataka 560067 India

Correction to: *Scientific Reports* 10.1038/s41598-024-61754-4, published online 13 May 2024

The Competing interests section in the original version of this Article was incorrect.

“The authors declare no competing interests.”

now reads:

“Dr Srikanth Sola and Radhica Kanniganti are named as inventors on the patent WO2021245687A1 titled "A system and a method for reducing particulate pollutants in air, using pulsed electromagnetic waves" (https://patents.google.com/patent/WO2021245687A1). They have received compensation as a member of the Devic Earth Private Limited research team. Dr G. S. N. V. K. S. N. Swamy Undi, Cheramangalath Balan Ramya & Kiran Shinde declare no potential conflict of interest.”

The original Article has been corrected.

